# Biomarker Profiles and Clinicopathological Features in Head and Neck Squamous Cell Carcinoma Patients

**DOI:** 10.3390/medicina60101681

**Published:** 2024-10-14

**Authors:** Timea Szatmari, Simona Mocan, Cristian Mircea Neagos, Zsuzsanna Pap

**Affiliations:** 1 The Doctoral School, George Emil Palade University of Medicine, Pharmacy, Sciences, and Technology of Targu Mureș, 540142 Targu Mures, Romania; 2 Department of Pathology, County Emergency Hospital, 540136 Targu Mures, Romania; 3 Department of Anatomy and Embryology, George Emil Palade University of Medicine, Pharmacy, Sciences, and Technology of Targu Mureș, 540142 Targu Mures, Romania

**Keywords:** head and neck squamous cell carcinoma, immunohistochemistry, EGFR, Ki67, p53, Cx43, COX-2, p16

## Abstract

*Background and Objectives:* Head and neck squamous cell carcinomas (HNSCCs) vary significantly in terms of invasiveness, growth rate, and metastatic potential. This study aimed to investigate the expression of several prognostic biomarkers (Ki67, p53, EGFR, COX-2, Cx43, and p16) in HNSCC from various anatomical regions and to correlate these expressions with clinicopathological parameters. *Materials and Methods*: We performed immunohistochemistry on 91 histologically verified HNSCC cases from the County Emergency Hospital, Targu Mures. Biomarker expression for Ki67, COX-2, and Cx43 was assessed using a standard immunoexpression scoring system: S1: 0–10%, S2: 11–25%, S3: 26–50%, S4 > 50%; EGFR was scored based on membrane staining intensity: 0, 1+, 2+, 3+; we classified p16 as positive or negative; p53 was grouped into mutant and wild-type; and we compared these across histopathological types, tumor grades, anatomical locations, gender, and different age groups. We performed a comparative analysis of Cx43 expression levels in relation to the expression of the rest of the markers. Statistical analysis was conducted using GraphPad InStat 3 software, version 3.06 (GraphPad Software Inc., San Diego, USA). *Results*: The majority of tumors were in males (95.6%) aged 51–60 years. Mutant p53 expression was prevalent in most cases. Elevated Ki67 and EGFR expression were associated with more aggressive tumors. COX-2 levels varied, with a higher proportion of moderate and high immunoexpression (S3 + S4) observed in patients under 70 years old. Cx43 expression was generally low, especially in extralaryngeal tumors. *Conclusions*: HNSCC primarily affects older males, with the larynx being the most common site. High levels of Ki-67 and EGFR suggest more aggressive tumors, while low COX-2 levels reflect varying prognoses. Women may develop more aggressive tumors, and extralaryngeal tumors often present with more challenging prognoses. Low Cx43 expression may be more likely to coincide with higher Ki67 and COX-2 levels, possibly indicating a link with more aggressive tumor behavior.

## 1. Introduction

Head and neck cancers are predominantly squamous cell carcinomas (SCC). However, tumors affecting different regions within this category can vary significantly in terms of invasiveness, growth rate, and metastatic potential [1]. To better understand the behavior of these tumors, researchers worldwide have been increasingly focusing on biomarker studies over the past few decades. Biomarkers play a critical role in improving the precision and effectiveness of cancer diagnosis, prediction of treatment response, and management [2].

In this study, we systematically examined the expressions of several published prognostic markers (Ki67, p53, EGFR, COX-2, Cx43) and p16 in head and neck squamous cell carcinomas from various anatomical regions using immunohistochemistry.

Reports indicate that HPV (human papillomavirus)-positive oropharyngeal SCC represents a distinct clinicopathological entity with a better prognosis compared to HPV-negative oropharyngeal SCC [3]. Multiple studies have shown that increased expression of p16 is an excellent surrogate marker for HPV-positive oropharyngeal SCC [4].

Ki-67 is a protein that is associated with cell proliferation. High Ki-67 levels in head and neck cancer are generally associated with more aggressive tumors and poorer prognosis. Sometimes, Ki-67 is used as part of the grading system for tumors, helping to guide treatment planning [5,6].

The p53 protein is crucial for DNA stability and cancer prevention. Its role in head and neck cancer is complex and context-dependent. As a tumor suppressor, p53 regulates the cell cycle, apoptosis, and genomic stability. High levels of normal p53 can indicate a positive prognosis by promoting apoptosis and DNA repair, aiding in cancer control and enhancing treatment effectiveness. Conversely, high p53 levels due to TP53 gene mutations result in stable, mutant p53 proteins, leading to uncontrolled growth, resistance to apoptosis, and genomic instability, often associated with more aggressive cancer and worse outcomes, including in HNSCC [7,8].

Epidermal growth factor receptor (EGFR) is a protein that, when overexpressed or mutated, can play a significant role in the development and progression of various cancers. High EGFR levels in HNSCC indicate poor prognosis, as EGFR promotes tumor growth, invasion, and metastasis. It serves as both a prognostic marker and a therapeutic target. EGFR inhibitors, such as monoclonal antibodies and tyrosine kinase inhibitors, are critical in treatment [9].

Cyclooxygenase-2 (COX-2) is an enzyme involved in the synthesis of prostaglandins, which play a role in inflammation and cell proliferation. COX-2 overexpression is associated with enhanced tumor growth, angiogenesis, and metastasis. Prostaglandins produced by COX-2 can promote these processes, contributing to the aggressiveness of the cancer. It is also considered both a prognostic marker and a potential therapeutic target in head and neck squamous cell carcinoma (HNSCC) [10].

Membrane connexin 43 (Cx43) forms gap junctions, which are channels enabling direct communication between adjacent cells. Experimental data indicate that gap junction proteins play a significant role in tumor growth and progression. These intercellular channels, composed of about 21 connexins, facilitate metabolic cooperation, growth, and development. The absence of gap junctions and intercellular communication can contribute to tumorigenesis by promoting cell migration, invasion, and metastasis [11]. Of the 21 connexins, Cx43 is the most well-known and extensively studied. It is broadly expressed in epithelial cells, hematopoietic cells, neurons, astrocytes, cardiac neural crest cells, and fibroblasts. Cx43 regulates cell proliferation and apoptosis by forming hemichannels that exchange growth and apoptotic factors, while also promoting tumor progression and metastasis. Recent studies suggest that Cx43 plays a more complex role in various stages of tumor progression [12,13]. Assessing Cx43 expression can help determine the aggressiveness of cancer, guide treatment strategies, and explore targeted therapies that could potentially restore its function or enhance its tumor-suppressive properties. Measuring Cx43 levels in tumor tissues could also have potential diagnostic use [14,15].

Aim: Our objective was to investigate how regional variations in the expression of potential biomarkers of SCC progression in the head and neck region contribute to understanding the diverse behaviors of these malignancies and to correlate results with clinicopathological parameters.

## 2. Materials and Methods

We performed immunohistochemistry on 91 histologically verified HNSCC cases [48 laryngeal (L), 25 extralaryngeal (E), 18 cases affecting multiple regions (E-L)] from 2010 to 2016 selected from the archive of the County Emergency Hospital, Targu Mures. The laryngeal group (L) included HNSCC from supraglottic, glottic, or subglottic areas; the extralaryngeal group (E) covered tumors in the nasopharynx, oropharynx, hypopharynx, oral cavity, and mobile tongue; and the mixed group (E-L) comprised cases with both laryngeal and extralaryngeal spread. We classified patients’ squamous cell carcinomas into three groups: classic (CSCC), variants (CSCV—basaloid, spindle cell, acantholytic, verrucous, lymphoepithelial, papillary, and adenosquamous), and mixed-type (CSCM—tumors with features of two histological types). CSCC included keratinized and non-keratinized tumors. We used the WHO-recommended three-tier grading system to grade tumors: G1 for well-differentiated, G2 for intermediate, and G3 for poorly differentiated tumors.

In the Immunohistochemistry Laboratory of the Department of Anatomy and Embryology at the “George Emil Palade” University of Medicine, Pharmacy, Science, and Technology, the 3 μm thick sections obtained from the formalin fixed and paraffin embedded resection tissue specimens were dewaxed and rehydrated followed by endogenous peroxidase blocking. Antigen retrieval was performed by pressurized steam cooking (citrate solution, pH 10 for p53, pH 6 for Cx43) or microwave treatment (pH 10 forKi67, EGFR, pH 6 for p16, COX-2). We used mouse monoclonal antibodies for p53 (clone DO-7, Novocastra Laboratories Ltd., Leica Biosystems, Deer Park, IL, USA) in 1/800, Ki67 (clone MM1, Novocastra Laboratories Ltd., Leica Biosystems, Deer Park, IL, USA) in 1/150, EGFR (clone EGFR.113, Novocastra Laboratories Ltd., Leica Biosystems, Deer Park, IL, USA) in 1/40, COX-2 (clone COX229, Thermo Fisher Scientific, Waltham, MA, USA) in 1/100 for 1 h, as well as Cx-43 (clone CX-1B1, Thermo Fisher Scientific, Waltham, MA, USA) in 1/100 (overnight, 4 °C). For p16, we applied rabbit clonal antibody (clone R19-D, DB Biotech, Inc., Kosice, Slovakia) in 1/200 (1 h). For signal amplification, the secondary antibody EnVision Flex/HRP (Horseradish peroxidase) (Dako, 20 min) was used. Detection of primary antibodies was achieved using 3,3′-Diaminobenzidine (DAB, Dako, Santa Clara, CA, USA). The slides were then counterstained with hematoxylin, dehydrated, and mounted. Negative controls were performed by omitting the primary antibody. Immunohistochemical reactions were read by an independent pathologist.

Nuclear expression was observed for p53 and Ki67 (only the infiltrative component on 1000 cells), while COX-2 displayed cytoplasmic expression. The expression was membranous for EGFR and Cx43. We interpreted the results for Ki67, COX-2, and Cx43 according to the following immunoexpression scoring system: S1: 0–10%, S2: 1–25%, S3: 26–50%, S4 > 50%. For p16, we classified the reaction as positive if at least 75% of the infiltrative tumor cells showed intense cytoplasmic and nuclear positivity, considering the remaining reactions as negative.

We classified p53 into two categories: mutant and non-mutant (wild-type). Three types of reactions were considered as mutants: negative nuclear reaction (no cells with a positive nuclear reaction), positive nuclear reaction (more than 90% of tumor cells present an intense, uniform nuclear reaction), and cytoplasmic reaction (tumor cells present an aberrant positive cytoplasmic reaction and not nuclear), while all other reactions were classified as wild-type.

EGFR was scored based on membrane staining intensity: 0 = no staining or <10% of tumor cells show membrane staining; 1+ = faint membrane staining in >10% of tumor cells; 2+ = moderate membranous staining for at least 10% of tumor cells; 3+ = >10% of tumor cells with strong membranous staining.

For statistical analysis, we used GraphPad InStat 3 software, version 3.06 (GraphPad Software Inc., San Diego, CA, USA). A significant association was taken into consideration at a *p* value of <0.05, with a 95% confidence interval.

Ethics. This study was approved by the ethics committee of “George Emil Palade” University of Medicine, Pharmacy, Science, and Technology of Târgu Mureș, Romania (no. 3211/10/06/2024, provided on 10 June 2024).

## 3. Results

Most of the tumors were seen in males (95.6%) aged between 51 to 60 (41.76%). The median age at diagnosis was 65 years (range: 39–81 years). Among the main histopathological groups observed, 74 cases (81%) were classified as classic squamous cell carcinoma (CSCC), 6 cases (7%) as variants (CSCV), and 11 cases (12%) as mixed-type (CSCM). Most tumors were classified as grade G2 (41 cases, 51.25%) or G3 (37 cases, 46.25%), with only 2.5% being well-differentiated (G1).

### 3.1. Ki67 Immunoexpression

Most CSCC cases showed S1 (40.62%) and S3 (32.81%) immunoexpression. In the variants, elevated Ki67 expression was observed (S3: 40%, S4: 40%), while in the mixed group, most cases were classified in the middle expression grades (S2: 33.33%, S3: 44.44%). Regarding tumor grading, there was minimal Ki67 expression in well-differentiated tumors. Intermediate differentiated grade tumors mostly showed S1 immunoexpression (51.42%), while poorly differentiated tumors predominantly exhibited S3 expression (35.48%). For tumors located in the L and E-L regions, most tumor cells exhibited S1 and S3 expression. In contrast, the majority of extralaryngeal tumors were S3 (54.54%) and S4 (27.27%). In men, most tumors exhibited S1 (37.83%) and S3 (33.78%) expression. On the other hand, in women, half of the cases were classified as S3 for Ki67 staining, with the remaining cases were equally divided between S2 and S4, and no cases were classified as S1. The ratio of cases with high Ki67 immunoexpression (S4) was higher in patients under 70 years old (29%, 22/75), compared to tumors developed in patients over 71 years old, where moderate immunoexpression (S3) was more common (5/10, 50%) (Table 1 and Table 2).

**Figure 1:**
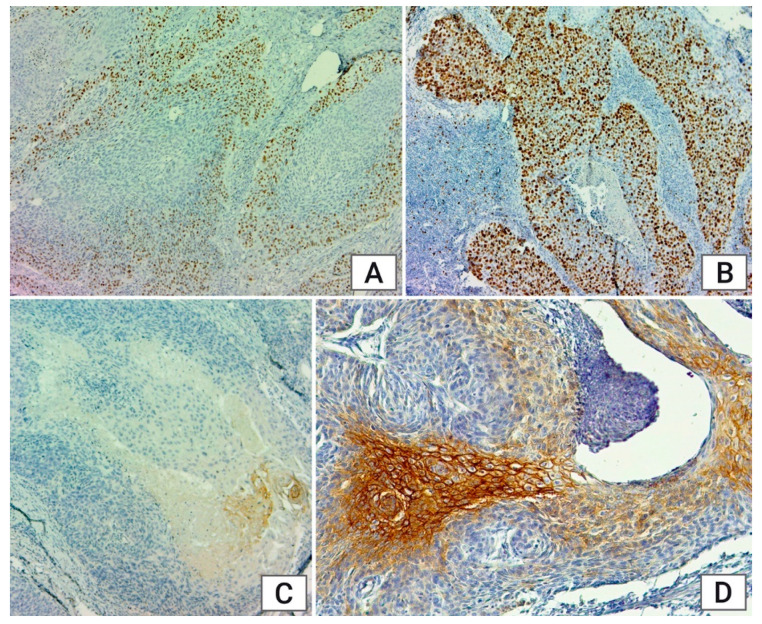
Immunohistochemical staining: (**A**): Ki 67 intense positive nuclear staining in less than 10% of tumor cells, ×4; (**B**): Ki 67: intense positive nuclear staining in more than 50% of tumor cells, ×4; (**C**): Cx43 membrane reaction of weak intensity in less than 10% of the tumor cells, ×4; (**D**): Cx43 membrane reaction of increase intensity in 20% of the tumor cells, ×4.

Ki67 expression correlated significantly with tumor localization (*p* = 0.01) and grade of the tumors (*p* = 0.048) (Figure 1A,B).

### 3.2. p53 Immunoexpression

**Figure 2:**
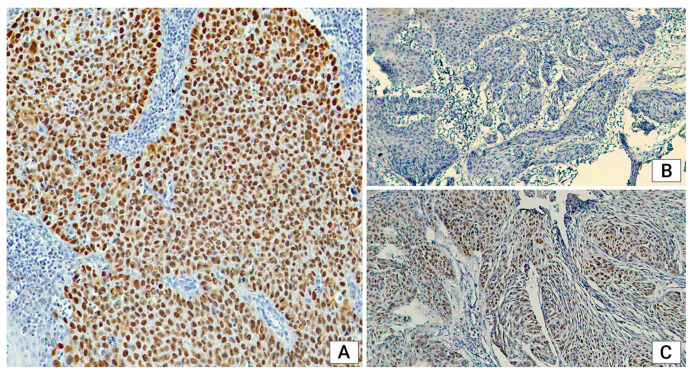
Immunohistochemical staining. (**A**): p53 aberrant mutational reaction, positive type, with over 90% of the tumor cells presenting a strong nuclear reaction, ×10; (**B**): p53 aberrant mutational reaction, negative type, with no nuclear reaction in tumor cells, ×10; (**C**): p53 normal wild-type expression, with nuclear reaction with variable intensity in some of the tumor cells, ×10.

For mutant p53 expression, we found 97.95% of cases with negative mutant reactions (0% cell staining) and 2.04% with positive mutant reactions (>90% cell staining); there was no cytoplasmic staining. When examining the localization, mutant p53 expression was substantially higher in both laryngeal and extralaryngeal tumors (L: 64.28%, E: 68.18%). While in the mixed group of tumors, the opposite was observed, with wild-type p53 expression being higher (E-L: 61.11%). Tumors from the CSCV group exclusively exhibited mutant p53 expression, which was also more prevalent in the CSCC malignancies (58.82%). Wild-type staining was higher only in the CSCM group (55.5%). Among women, all cases showed mutant p53 expression, while 58.22% of cases in men were classified similarly. The results showed mutant p53 expression across all tumor grades, including well-differentiated, intermediate, and poorly differentiated tumors. The ratio of cases with mutant p53 expression was higher in patients under 60 years old (32/45, 71.11%) compared to patients over 61 years old (17/37, 45.94%). p53 expression demonstrated a statistically significant correlation with the patients age (Figure 2A–C).

### 3.3. p16 Immunoexpression

In the analysis of all the factors, low p16 expression was observed in the majority of tumors, and based on the positivity criteria, p16 expression did not reach a positive level in any case.

### 3.4. EGFR Immunoexpression

**Figure 3:**
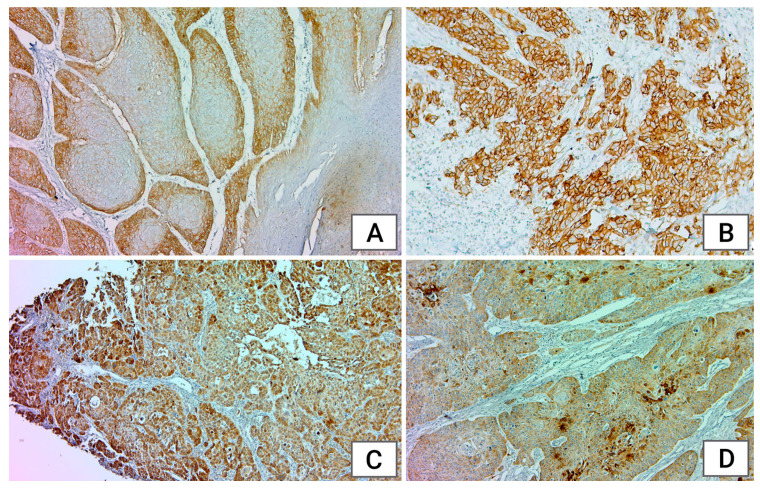
Immunohistochemical staining: (**A**): EGFR score 2+ with moderate membranous staining for at least 10% of tumor cells, ×4; (**B**): EGFR score 3+ with almost 100% of tumor cells with strong membranous staining, ×4; (**C**): COX-2: cytoplasmic positive and intense expressions in more than 50% of squamous cell carcinoma, ×4; (**D**): COX-2: cytoplasmic immunohistochemical reaction with variable intensity, ×4.

Among the histological types, most CSCC (33.33%) and CSCV (50%) tumors showed a 2+ reaction, while CSCM tumors exhibited weak (1+: 33.33%) or no (0+: 33.33%) expression in most cases. In poorly differentiated tumors, EGFR expression was most evenly distributed (0+: 35.48%, 1+: 22.58%, 2+: 19.35%, 3+: 22.58%), likewise in the L region (0+: 25.64%, 1+: 28.20%, 2+: 25.64%, 3+: 20.51%). The same pattern was observed when examining cases separately for women and men. In both the youngest and oldest patients, 1+ expression was the most common; whereas, in the 51–60 and 61–70 age groups, 2+ and 3+ expression were more prevalent. EGFR expression does not show statistically significant correlations with localization, histology type, age, or gender (*p* > 0.05) (Figure 3A,B).

### 3.5. COX-2 Immunoexpression

Studying COX-2 expression across different histological types, we found that S1, S3, and S4 expression occurred at similar rates in all three groups; whereas, S2 expression was observed in fewer tumors (CSCC: 7.35%, CSCV: 0%, CSCM: 0%). Most tumors exhibited S3 expression across all anatomical regions (L: 33.33%, E: 47.61%, E-L: 25.29%). The tumor grading was directly proportional to the immunoexpression score. Women exhibited either weak or increased staining, while in men, the distribution was more balanced (S1: 28%, S2: 8%, S3: 38.66%, S4: 25.33%). The ratio of cases with moderate and high immunoexpression (S3 + S4) was higher in patients under 70 years old (45/67, 67.16%) compared to those over 71 years old, where low immunoexpression (S1) was more prevalent (5/10, 50%) (Figure 3C,D).

### 3.6. Cx-43 Immunoexpression

Cx-43 expression in most CSCC (85.29%) and CSCV (100%) tumors was S1. In CSCM tumors, the distribution was more balanced, with S1 at 72.72%, S2 at 18.18%, and S3 at 9.09%, but no cases had more than 51% expression. Regarding tumor grading, the number of tumors with S1 expression decreased as the tumors became less differentiated, while an increase in S2 expression was observed with poorer differentiation levels. Considering all anatomical regions, low-expression tumors were the most common everywhere (L:39.06%, E:95.45%, E-L 68.18%). In the age groups, we observed that while most tumors exhibited low expression, the number of tumors showing S2 and S3 expression was similar across all age groups. In men, 83.75% of tumors had S1 staining, while in women, no tumors exhibited expression greater than S1 immunoexpression (11%) (Figure 1C,D).

### 3.7. Comparative Analysis of Cx43 Expression Levels

**Figure 4:**
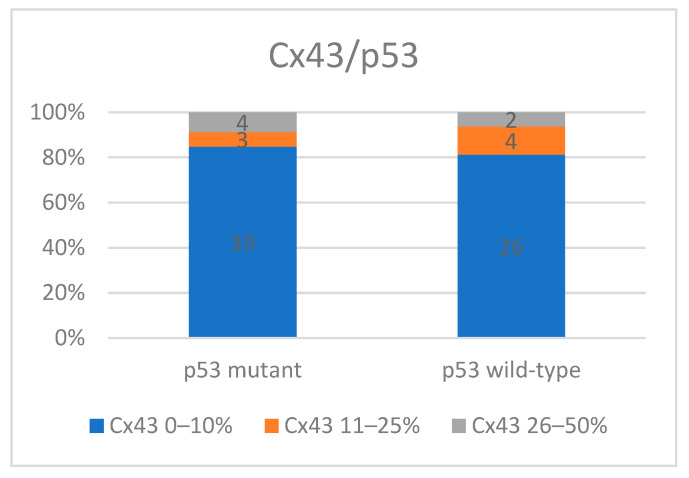
Cx43 expression in relation to p53 expression.

We performed a comparative analysis of Cx43 expression levels in relation to the expression of Ki67, COX-2, and EGFR, particularly in the context of p53 mutation status. When evaluating Cx43 expression levels with p53 status, it appeared that the majority of cases in both groups exhibit low Cx43 expression (S1). In the case of tumors with mutant p53 along with Cx43 S1 immunoexpression (84.7%), the most frequent were cases with Cx43 S3 immunoexpression (8.7%), while in wild-type tumors, S2 was more common (S1: 81.2%, S2: 12.5%) (*p* = 0.63) (Figure 4).

**Figure 5:**
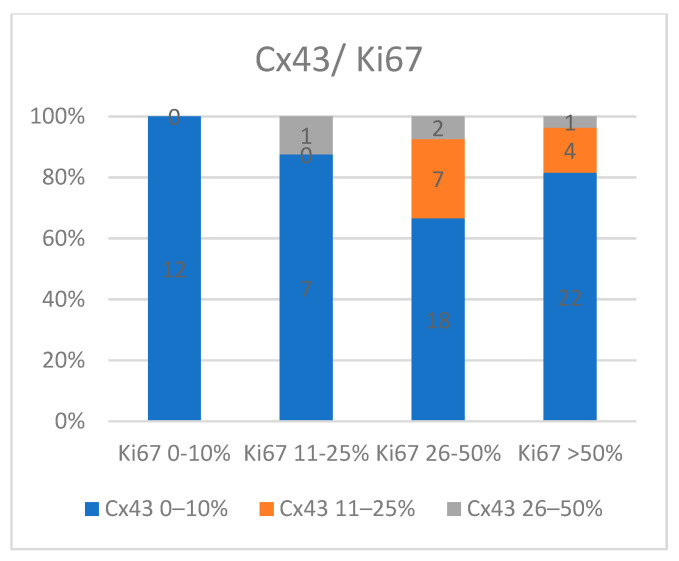
The relationship between Cx43 and Ki67 expression.

Comparing Ki67 immunoexpression with Cx43, we observed that all cases with Ki67 S1 immunoexpression were associated with Cx43 S1 expression (*p* = 0.16). The number of cases with partially preserved Cx43 immunoexpression increased with higher Ki67 levels. However, these cases still exhibited relatively high Ki67 expression (S3 and S4), indicating that moderate Cx43 expression might still be associated with higher proliferation rates (Figure 5).

**Figure 6:**
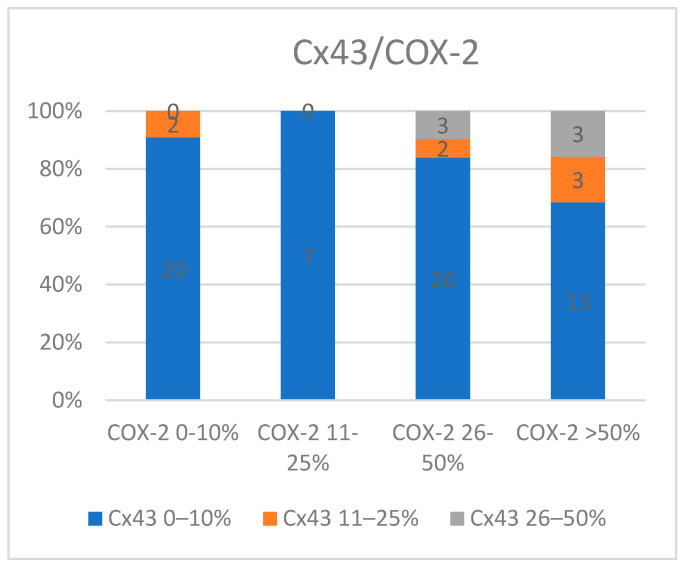
The relationship between Cx43 and COX-2 expression.

The data suggest that low Cx43 expression (S1) is common and is associated with a wide range of COX-2 expression. Cases with COX-2 S1 immunoexpression were more frequently associated with Cx43 S1 immunoexpression (93.1%) compared to those with COX-2 immunoexpression greater than 26% (78%). This pattern indicates that moderate Cx43 expression might be linked to higher levels of COX-2 activity (*p* = 0.12) (Figure 6).

**Figure 7:**
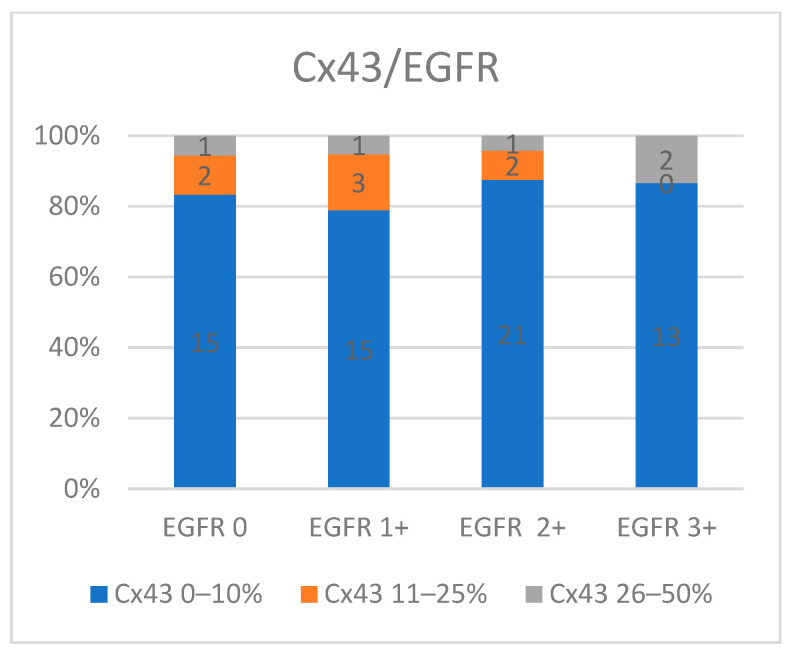
Cx43 expression in association with EGFR expression.

When evaluating the EGFR expression, we found that is widely distributed across all levels (0 to 3+) within the S1 expression level of Cx43. Tumors with EGFR 2+ and 3+ expression are more frequently associated with Cx43 S1 expression (87.1%) compared to those with EGFR 0 and 1+ expression (81%). As Cx43 expression increases to moderate levels, the number of cases declines, but EGFR expression remains varied (*p* = 0.43) (Figure 7).

The relationship between Cx43 expression and other markers (Ki67, COX-2, EGFR, p53) varies and is complex. Low Cx43 expression is common across all groups and does not show a strong correlation with the other markers. However, low Cx43 expression may be more likely to coincide with higher Ki67 and COX-2 levels, possibly indicating a link with more aggressive tumor behavior. EGFR expression, however, appears to be independent of Cx43 levels.

## 4. Discussion

The incidence of head and neck cancers continues to show an increasing trend worldwide. In our study population, 95% of the patients were male, with most aged between 51 and 60 years; although, the number of female patients is also rising [16]. HNSCCs are a diverse, aggressive, and genetically complex group of malignancies. For decades, the prognosis of tumors and their expected response to different treatments have mainly been predicted using the TNM classification. In recent years, many studies have explored the importance of various immunohistochemical markers and their clinical utility in offering more precise and personalized prognoses.

Across the range of markers studied, one of the most promising is EGFR, with its overexpression or aberrant activation potentially leading to uncontrolled cell growth and tumor development [17]. We observed milder EGFR expression in both younger and older patients, while those aged 50–70 exhibited higher expression levels, which may indicate more aggressive tumor growth and a poorer prognosis in this age group, as reflected in the literature [3,18].

In the analysis of all factors, low p16 expression was observed in most tumors, not reaching the positivity criteria in any case. This largely aligns with the literature, which indicates that p16 positivity is less likely to be expected of tumors that originated from or involved the larynx. Additionally, none of the tumors diagnosed in extralaryngeal regions showed positive p16 expression [19,20,21].

Ki67 levels provide insights into cancer proliferative activity and significantly correlate with tumor grade (*p* = 0.048). Well-differentiated tumors showed minimal Ki67 expression, intermediate-grade tumors mostly exhibited S1 expression (51.42%), and poorly differentiated tumors predominantly had S3 expression (35.48%). This finding supports existing literature, which indicates that higher Ki67 expression is associated with a greater likelihood of poorly differentiated, poorer-prognosis tumors [6]. We observed higher Ki67 expression in extralaryngeal tumors compared to tumors originating from or involving the larynx, with a significant correlation based on tumor localization (*p* = 0.01).

Examining p53 expression in relation to various factors, we observed that mutant p53 expression was prevalent in most cases, indicating a worse prognosis. Specifically, younger patients (under 60 years) had a higher ratio of tumors with mutant p53 expression. This correlation with age was statistically significant (*p* = 0.02), suggesting that tumors in younger individuals may be more aggressive and associated with a worse prognosis, reflecting a more aggressive tumor phenotype. The gender distribution analysis revealed that all tumors in women exhibited mutant p53 expression, indicating a worse prognosis compared to men and suggesting the need for more complex therapeutic solutions. The majority of cases with mutant p53 expression had negative mutant reactions with 0% cell staining. Currently, the literature does not emphasize whether mutant p53 expression is classified as negative or positive. Wild-type p53 expression was observed only in histopathologically mixed-type tumors, suggesting that tumors with the features of two histological types may be more aggressive [22].

No significant differences in COX-2 expression were observed across different tumor localizations, with most anatomical regions showing S3 expression, which means the level of COX-2 did not vary much depending on where the tumor was located. The tumor grading was directly proportional to the immunoexpression score. Knowing that high COX-2 levels indicate cancer aggressiveness, this suggests that more aggressive or targeted treatment strategies may be necessary to manage these cases effectively. The ratio of cases with moderate and high immunoexpression (S3 + S4) was higher in younger patients (under 70 years old: 67.16%) compared to those over 71 years old (50%), where low immunoexpression (S1) was more prevalent, suggesting the presence of less aggressive tumors with advancing age [23,24].

Cx43 is generally considered to have a tumor-suppressive function. It often has reduced expression or altered function in cancers such as HNSCC, leading to impaired cell communication and uncontrolled cancer cell growth, thus contributing to cancer progression [14]. Low-expression tumors were most common across all anatomical regions, with extralaryngeal tumors showing especially high rates of low expression, where 95.45% exhibited S1 expression. This may indicate that tumors in the extralaryngeal region have a poorer prognosis in the studied population. In men, 16.25% of the tumors exhibited stronger expression (S2 + S3); whereas in women, all tumors had low expression. According to the literature, this may suggest that women could develop more aggressive head and neck tumors with a poorer prognosis [25].

## 5. Conclusions

The present study highlights that HNSCCs are predominant in older males, with the larynx being the most common site. Our study explored the relationship between histological and clinical parameters and biomarker profiles in HNSCC. High levels of EGFR and Ki67 expression are linked with more aggressive tumors, while low p16 expression and COX-2 levels suggest varying prognoses based on tumor localization and age. Additionally, the findings also suggests that women may develop more aggressive tumors with poorer outcomes, and extralaryngeal tumors often have a more challenging prognosis. Despite progress in understanding HNSCC, finding a consistently reliable biomarker profile is still ongoing. A definitive profile could enhance diagnosis, prognosis, and treatment, but more research and validation are needed.

## Figures and Tables

**Table 1:** Immunoexpression of p53, p16, ki67, COX-2, CX-43, EGFR in relation to histopathological type (CSCC: classic squamous cell carcinomas, CSCV: variants, CSCM: mixed-type), tumor grading (G1: well-differentiated, G2: intermediate, G3: poorly differentiated) and localization (L: laryngeal, E: extralaryngeal, E-L: mixed group).

p53	CSCC	CSCV	CSCM	G1	G2	G3	L	E	E-L
Mutant	40	5	4	1	24	18	27	15	7
Wild-type	28	0	5	0	13	16	15	7	11
*p*		*p* = 0.11			*p* = 0.42			*p* = 0.11	
**Ki67**	**CSCC**	**CSCV**	**CSCM**	**G1**	**G2**	**G3**	**L**	**E**	**E-L**
0–10%	26	1	1	0	18	8	4	1	7
11–25%	5	0	3	1	3	4	10	3	1
26–50%	21	2	4	0	10	11	4	12	5
>51%	12	2	1	0	4	8		6	4
*p*		*p* = 0.14			*p* = 0.048			*p* = 0.01	
**COX-2**	**CSCC**	**CSCV**	**CSCM**	**G1**	**G2**	**G3**	**L**	**E**	**E-L**
0–10%	18	1	3	1	14	5	12	7	3
11–25%	6	0	0	0	3	3	3	2	1
26–50%	23	1	5	0	11	14	13	10	6
>51%	16	1	3	0	7	11	11	2	7
*p*		*p* = 0.94			*p* = 0.25			*p* = 0.46	
**Cx43**	**CSCC**	**CSCV**	**CSCM**	**G1**	**G2**	**G3**	**L**	**E**	**E-L**
0–10%	58	4	8	2	31	30	34	21	15
11–25%	5	0	2	0	3	3	5	1	1
26–50%	5	0	1	0	5	1	4	0	2
>51%	0	0	0	0	0	0	0	0	0
*p*		*p* = 0.67			*p* = 0.59			*p* = 0.43	
**EGFR**	**CSCC**	**CSCV**	**CSCM**	**G1**	**G2**	**G3**	**L**	**E**	**E-L**
0	14	1	3	0	6	11	10	5	3
1+	16	0	3	0	9	7	11	4	4
2+	21	2	1	1	14	6	10	11	3
3+	12	1	2	0	6	7	8	1	6
*p*		*p* = 0.75			*p* = 0.32			*p* = 0.13	

**Table 2:** Immunoexpression of p53, p16, ki67, COX-2, Cx43, EGFR in relation to gender and age groups.

p53	Female	Male	30–50	51–60	61–70	>71 Years
0–10%	3	46	4	28	12	5
11–25%	0	33	5	8	16	4
*p*	*p* = 0.14			*p* = 0.02		
**Ki67**						
0–10%	0	28	2	12	10	4
11–25%	1	7	2	2	3	1
26–50%	2	25	3	11	8	5
>51%	1	14	2	12	8	0
*p*	*p* = 0.42			*p* = 0.6		
**COX-2**						
0–10%	1	21	1	10	6	5
11–25%	0	6	1	2	2	1
26–50%	0	29	3	16	8	2
>51%	1	19	2	6	10	2
*p*	*p* = 0.63			*p* = 0.57		
**Cx43**						
0–10%	3	67	7	29	25	9
11–25%	0	7	1	2	3	1
26–50%	0	6	1	3	1	1
>51%	0	0	0	0	0	0
*p*	*p* = 0.74			*p* = 0.96		
**EGFR**						
0	1	17	1	11	4	2
1+	1	18	4	5	6	4
2+	1	23	1	12	9	2
3+	1	14	0	10	3	2
*p*	*p* = 0.98			*p* = 0.21		

## Data Availability

All data here are available upon request.

## References

[1] Lukits J., Timár J., Juhász A., Döme B., Paku S., Répássy G. (2001). Progression difference between cancers of the larynx and hypopharynx is not due to tumor size and vascularization. Otolaryngol. Head. Neck Surg..

[2] Becker A.S., Kluge C., Schofeld C., Zimpfer A.H., Schneider B., Strüder D., Redwanz C., Ribbat-Idel J., Idel C., Maletzki C. (2023). Identifying Predictive Biomarkers for Head and Neck Squamous Cell Carcinoma Response. Cancers.

[3] Ang K.K., Harris J., Wheeler R., Weber R., Rosenthal D.I., Nguyen-Tân P.F., Westra W.H., Chung C.H., Jordan R.C., Lu C. (2010). Human papillomavirus and survival of patients with oropharyngeal cancer. N. Engl. J. Med..

[4] Shi W., Kato H., Perez-Ordonez B., Pintilie M., Huang S., Hui A., O’Sullivan B., Waldron J., Cummings B., Kim J. (2009). Comparative prognostic value of HPV16 E6 mRNA compared with in situ hybridization for human oropharyngeal squamous carcinoma. J. Clin. Oncol..

[5] Menon S.S., Guruvayoorappan C., Sakthivel K.M., Rasmi R.R. (2019). Ki-67 protein as a tumour proliferation marker. Clin. Chim. Acta.

[6] Li L.T., Jiang G., Chen Q., Zheng J.N. (2015). Ki67 is a promising molecular target in the diagnosis of cancer (review). Mol. Med. Rep..

[7] Mireștean C.C., Iancu R.I., Iancu D.P.T. (2022). p53 Modulates Radiosensitivity in Head and Neck Cancers-From Classic to Future Horizons. Diagnostics.

[8] Lees A., Sessler T., McDade S. (2021). Dying to Survive-The p53 Paradox. Cancers.

[9] Hashmi A.A., Hussain Z.F., Aijaz S., Irfan M., Khan E.Y., Naz S., Faridi N., Khan A., Edhi M.M. (2018). Immunohistochemical expression of epidermal growth factor receptor (EGFR) in South Asian head and neck squamous cell carcinoma: Association with various risk factors and clinico-pathologic and prognostic parameters. World J. Surg. Oncol..

[10] Frejborg E., Salo T., Salem A. (2020). Role of Cyclooxygenase-2 in Head and Neck Tumorigenesis. Int. J. Mol. Sci..

[11] Cronier L., Crespin S., Strale P.O., Defamie N., Mesnil M. (2009). Gap junctions and cancer: New functions for an old story. Antioxid. Redox Sign..

[12] Sirnes S., Lind G.E., Bruun J., Fykerud T.A., Mesnil M., Lothe R.A., Rivedal E., Kolberg M., Leithe E. (2015). Connexins in colorectal cancer pathogenesis. Int. J. Cancer.

[13] Matsuuchi L., Naus C.C. (2013). Gap junction Proteins on the move: Connexins, the Cytoskeleton and Migration. Biochim. Biophys. Acta BBA-Biomembr..

[14] Dános K., Brauswetter D., Birtalan E., Pató A., Bencsik G., Krenács T., Peták I., Tamás L. (2016). The Potential Prognostic Value of Connexin 43 Expression in Head and Neck Squamous Cell Carcinomas. Appl. Immunohistochem. Mol. Morphol..

[15] Brockmeyer P., Jung K., Perske C., Schliephake H., Hemmerlein B. (2014). Membrane connexin 43 acts as an independent prognostic marker in oral squamous cell carcinoma. Int. J. Oncol..

[16] Warnakulasuriya S. (2009). Global epidemiology of oral and oropharyngeal cancer. Oral Oncol..

[17] Bossi P., Resteghini C., Paielli N., Licitra L., Pilotti S., Perrone F. (2016). Prognostic and predictive value of EGFR in head and neck squamous cell carcinoma. Oncotarget.

[18] Byeon H.K., Ku M., Yang J. (2019). Beyond EGFR inhibition: Multilateral combat strategies to stop the progression of head and neck cancer. Exp. Mol. Med..

[19] Ramesh P.S., Devegowda D., Singh A., Thimmulappa R.K. (2020). NRF2, p53, and p16: Predictive biomarkers to stratify human papillomavirus associated head and neck cancer patients for de-escalation of cancer therapy. Crit. Rev. Oncol. Hematol..

[20] Katirachi S.K., Grønlund M.P., Jakobsen K.K., Grønhøj C., von Buchwald C. (2023). The Prevalence of HPV in Oral Cavity Squamous Cell Carcinoma. Viruses.

[21] Young D., Xiao C.C., Murphy B., Moore M., Fakhry C., Day T.A. (2015). Increase in head and neck cancer in younger patients due to human papillomavirus (HPV). Oral Oncol..

[22] Trovato M.C., Ruggeri R.M., Guzzo E., Certo R., Alibrandi A., Scifo S., Scardigno M., Vitarelli E., Arena G., Gambadoro O. (2017). Expression of P53 and isoforms in bening and malignant lesions of the head and neck. Histol. Histopathol..

[23] Yang B., Jia L., Guo Q., Ren H., Hu Y., Xie T. (2016). Clinicopathological and prognostic significance of cyclooxygenase-2 expression in head and neck cancer: A meta-analysis. Oncotarget.

[24] Mendes R.A., Carvalho J.F., Waal I.V. (2009). An overview on the expression of cyclooxygenase-2 in tumors of the head and neck. Oral Oncol..

[25] Puzzo L., Caltabiano R., Parenti R., Trapasso S., Allegra E. (2016). Connexin 43 (Cx43) Expression in Laryngeal Squamous Cell Carcinomas: Preliminary Data on Its Possible Prognostic Role. Head Neck Pathol..

